# Application of a battery of biotests for the determination of leachate toxicity to bacteria and invertebrates from sewage sludge-amended soil

**DOI:** 10.1007/s11356-012-1268-3

**Published:** 2012-11-07

**Authors:** Anna Malara, Patryk Oleszczuk

**Affiliations:** Faculty of Chemistry, Maria Curie-Skłodowska University, 3 Maria Curie-Skłodowska Square, 20-031 Lublin, Poland

**Keywords:** Sewage sludges, Toxicity, Bioassays, Biotests, Ecotoxicology

## Abstract

The objective of the study was to determine the leachates toxicity from sewage sludge-amended soils (sandy and loamy). Samples originated from a plot experiment realized over a period of 29 months. Two types of soil were fertilized with sewage sludges at the dose of 3 % (90 t/ha). Soil samples were taken after 0, 7, 17, and 29 months from the application of sewage sludges. Leachates were obtained according to the EN 12457–2 protocol. The following commercial tests were applied for the estimation of the toxicity: Microtox (*Vibrio fischeri*), Microbial assay for toxic risk assessment (ten bacteria and one yeast), Protoxkit F™ (*Tetrahymena thermophila*), Rotoxkit F™ (*Brachionus calyciflorus*), and Daphtoxkit F™ (*Daphnia magna*). The test organisms displayed varied toxicity with relation to the soils amended with sewage sludges. The toxicity of the leachates depended both on the soil type and on the kind of sewage sludge applied. Notable differences were also observed in the sensitivity of the test organisms to the presence of sewage sludge in the soil. The highest sensitivity was a characteristic of *B. calyciflorus*, while the lowest sensitivity to the presence of the sludges was revealed by the protozoa *T. thermophila*. Throughout the periods of the study, constant variations of toxicity were observed for most of the test organisms. The intensity as well as the range of those variations depended both on the kind of test organism and on the kind of sludge and soil type. In most cases, an increase of the toxicity of soils amended with the sewage sludges was observed after 29 months of the experiment.

**Electronic supplementary material:**

The online version of this article (doi:10.1007/s11356-012-1268-3) contains supplementary material, which is available to authorized users.

## Introduction

Sewage sludges are an interesting alternative to conventional fertilizers. Thanks to their content of nutrients and organic matter that they used for the improvement of the properties of soils and for soil reclamation (Epstein 2003). At the same time, natural utilization of sewage sludges involves the removal of troublesome wastes, the amounts of which increase year to year. However, due to their origins, sewage sludges may contain pollutants of toxic nature (Harrison et al. 2006). Research reveals that apart from contaminants that are already identified and more and more frequently analyzed, such as heavy metals, polycyclic aromatic hydrocarbons, polychlorinated biphenyls, dioxins, and furans (Harrison et al. 2006; Oleszczuk 2006; Smith 2009), sewage sludges may also contain new emerging contaminants (Díaz-Cruz et al. 2009; Muller et al. 2006; Smith 2009). Those newly identified contaminants are not determined on a routine basis during the estimation of sewage sludge applicability as fertilizers, in spite of the fact that they may pose a serious threat to the environment. Determination of those new contaminants often requires costly equipment that is available only at few laboratories, and still, there is no guarantee of achieving a complete estimate of threats related with their presence in sewage sludges. Therefore, it is necessary to search for solutions that will permit the identification of risks involved in the utilization of sewage sludges while permitting such assays to be conducted on a larger scale. In this aspect, estimation of sewage sludges with the use of biological tests may provide a significant complement to analytical studies (Gejlsbjerg et al. 2001; Natal-da-luz et al. 2009b; Oleszczuk 2008, 2010; Ramirez et al. 2008b). Biological tests constitute an interesting alternative to chemical analyses. They provide full representation of threats resulting not only from the presence of the contaminants but also from the properties of sewage sludges that may have a significant effect on the toxicity of the sludges. In addition, ecotoxicological estimation permits also the determination of toxicity resulting from potential interactions among the particular contaminants that appear in sewage sludges, which is something that cannot be achieved with the use of instrumental methods alone.

Issues concerning the toxicity of sewage sludges are a frequent subject of research (Fuentes et al. 2006; Hu and Yuan 2012; Oleszczuk 2008, 2010; Ramirez et al. 2008a, b; Roig et al. 2012). Those studies, however, are focused primarily on estimation of sludges as such, without taking into account other important parameters, e.g., the type of soil in which sewage sludge is applied. Also, studies conducted so far do not provide an answer to the question what is happening with the toxicity of sewage sludges after their application in soils over longer periods of time. It is a known fact that the type of soil may significantly determine the mobility and bioavailability of contaminants and, indirectly, also their toxicity (Domene et al. 2011). Therefore, it is important to conduct estimations of the toxicity of various soils amended with sewage sludges, in order to determine the potential risks involved in their application as fertilizers. With the passage of time, contaminants responsible for the toxic effect can undergo various processes (degradation, leaching, accumulation in plants, and sequestration), which may reduce their negative effect on organisms. There is also a possibility, e.g., as a result of mineralization of organic matter, of an increase in the bioavailability of contaminants, which, over time, can cause an intensification of the toxic effect (Oleszczuk 2009). So far, studies taking into account the above issues have not been undertaken, especially with relation to a broad group of organisms. In recent years, many new tests have appeared on the market, covering an extensive group of organisms. The application of a battery of those tests permits the acquisition of a complete representation of potential toxicity and the determination of potential risk for various groups of organisms (Matejczyk et al. 2011). Despite of the obvious advantages regarding biotests, there are also disadvantages, e.g., the lack of ecological relevance.

The objective of the study presented here was to determine the toxicity of leachates from two different soils (sandy and loamy) fertilized with sewage sludges of varied toxicity. The experiment was realized over a period of 29 months. The toxicity was studied with the use of commonly available ecotoxicological tests. Various groups of organisms (bacteria and invertebrates) were used in the study for the estimation of the potential effect of the leachates on aquatic organisms.

## Materials and methods

### Field experiment

The experiment was realized on two blocks of plots situated on different soils [sandy (soil S) and loamy (soil L)] characterized by varied physicochemical properties (Table 1) and origin (S—Haplic Podzol—originating from sand; L—Haplic Luvisol—originating from silt). The sandy soil was characterized by a strongly acidic pH, high content of H^+^ ions, and low content of carbon and nitrogen, while the loamy soil showed markedly better cultivation properties and was characterized by a neutral pH, good sorption qualities [cation exchange capacity (CEC)], and twice the nitrogen content of the soil S. The organic carbon content in the soil L was only slightly higher than that in soil S (Table 1). The soils differed also in their content of available forms of phosphorus (P^+^), potassium (K^+^), and magnesium (Mg^2+^). The greatest differences between the test soils were noted for the available forms of P^+^, followed by Mg^2+^. Soil S was characterized by a lower content of K^+^, but the differences were not as high as those observed for P and Mg^2+^ (Table 1).

**Table 1:** Physicochemical properties of soils and sewage sludges used in the experiment

Properties	Unit	Soils		Sewage sludges	
		S	L	SL1	SL2
Clay	%	2	9	–	–
Silt	%	8	75	–	–
Sand	%	90	16	–	–
pH	in KCl	3.6	7.1	6.1	7.6
CEC	mmol kg^−1^	20	190	440	976
TOC	g kg^−1^	8.3	10.7	188.2	157.2
N_t_	g kg^−1^	0.8	1.5	40.6	22.1
P_2_O_5_	mg/100 g soil	7.1	54.4	1,285.2	1,384.0
K_2_O	mg/100 g soil	14.7	26.0	2,623.9	2,390.3
Mg	mg/100 g soil	2.7	10.8	586.7	533.0

*S* sandy soil, *L* loamy soil, *SL1 and SL2* sewage sludges, *pH* reactivity, *CEC* cation exchange capacity, *TOC* total organic carbon content, *N*_*t*_ total nitrogen content, *P*_*2*_ *O*_*5*_ *, K*_*2*_ *O, and Mg* available forms of phosphorous, potassium, and magnesium, respectively

Sewage sludges (SL1 or SL2) were introduced into each soil once, at the dose of 90 t/ha. The dose has been chosen regarding to the Polish regulations. Sludge doses were calculated taking into consideration the sludge’s dry mass and the density of the solid soil phase. The sludge was mixed with the surface horizon of the soil (0–20 cm), and the substrate prepared in that manner was sown with a grass mix (*Lolium perenne*, *Festuca pratensis*, *Lolium multiflorum*, and *Phleum pratense*). The sewage sludges came from two different sewage treatment plants situated in southeast Poland, which purify predominantly municipal wastewater. The sewage sludges were collected at the end point of the sewage sludge digestion process. Sewage sludges were typical aerobically digested. The selected treatment plants were characterized by their catchment area (indicated by the number of inhabitants and amount of sewage treated) as well as by the industrial character of the area (quantity and variety of industrial plants). As in the case of the soils, the sewage sludges differed in most of their parameters. Compared to sludge SL2, sludge SL1 was characterized by a lower CEC value and pH. In sludge SL2, lower levels of organic carbon and nitrogen were noted, by 16.5 and 45.6 %, respectively, as compared to their content in sludge SL1.

The field experiment was carried out for 29 months. Soil samples were collected for analysis after the introduction of sewage sludge and then after 7, 19, and 29 months. Control soil (non-amended) and sewage sludge-amended soil samples were collected from the level of 0–20 cm with a (5–60 cm i.d.) stainless steel corer. Six independent samples (pseudo-replicates) were taken from each plot. The samples were transported to the laboratory, air dried in air-conditioned storage rooms (about 25 °C) for several weeks (in darkness), manually crushed, and sieved (<2 mm) prior to chemical analyses.

### Battery of biotests

Leachates from sewage sludge, soil, and sewage sludge-amended soil samples were obtained according to the EN 12457–2 protocol (EC 2002). The samples were mixed with deionized water in a single-stage batch test performed at a liquid-to-solid (L/S) ratio of 1 L/100 g. The glass bottles were shaken in a roller-rotating device at 10 rpm. The leachates were filtered by filter with a porosity of 0.45 μm.

A battery of five bioassays was used for this study: Microtox, microbial assay for toxic risk assessment (MARA), Protoxkit F™, Rotoxkit F™, and Daphtoxkit F™. Microtox reagents were purchased from SDI (Delaware, USA); MARA reagents, from NCIMB Ltd. (Aberdeen, UK), while microbiotests (Protoxkit F™, Rotoxkit F™, and Daphtoxkit F™) with all reagents and equipment were purchased from Microbiotests (Creasel, Deinze, Belgium).

The Microtox® toxicity test was used to evaluate the inhibition of the luminescence in the marine bacteria *Vibrio fischeri* according to the test protocol (SDI 1992). The tests were carried out using a Microtox M500 analyzer. The light output of the luminescent bacteria from sewage sludge or soil’s extract was compared with the light output of a blank control sample. Luminescence inhibition of extract was assessed for 15 min of exposure carrying out the “81.9 % basic test protocol” (screening test; MicrotoxOmni software was used).

Microbial assay for risk assessment is a multispecies assay which allows measurement of toxic effects of chemicals and environmental samples. The test uses a selection of taxonomically diverse microbial species lyophilized in a microplate. Ten prokaryotic species and a eukaryote (yeast) constitute the biological indicators of toxicity assessment. In the present work, the microbial species used consisted of ten bacterial species: (1) *Microbacterium* sp., (2) *Brevundimonas diminuta*, (3) *Citrobacter freundii*, (4) *Comamonas testosteroni*, (5) *Enterococcus casseliflavus*, (6) *Delftia acidovorans*, (7) *Kurthia gibsonii*, (8) *Staphylococcus warnerii*, (9) *Pseudomonas aurantiaca*, and (10) *Serratia rubidaea*, and one yeast species, (11) *Pichia anomala* (Wadhia et al. 2007). The growth of the organisms exposed to the test sample is determined with the reduction of tetrazolium red. A scanned image of the microplate obtained using a flatbed scanner is analyzed using Purpose Built software. The MARA test was performed according to the standard protocol described by Wadhia et al. (2007).

Protoxkit F™ test measures growth inhibition of the ciliate protozoan *Tetrahymena thermophila* after 24 h. The tests were performed according to the standard operational procedure manual of the Protoxkit F™ (Protoxkit 1998).

Twenty-four-hour toxicity tests with *Brachionus calyciflorus* were conducted using the Rotoxkit F™. The test was carried out according to the manufacturer’s instructions (Rotoxkit 2000). Test plates inoculated with *B. calyciflorus* were incubated at 25 °C in the dark, and immobilization of *B. calyciflorus* after exposure to the toxicants was used as a toxicity end point.

Daphtoxkit F™ acute test with *Daphnia magna* makes use of neonates hatched from dormant eggs (ephippia) to determine the inhibition of motility after 24 h. Tests were performed according to the standard operational procedure manual of the Daphtoxkit F™ magna (Daphtoxkit 1996), which follows OECD Guideline 202 (OECD 1984).

### Physicochemical properties

The chemical properties of sewage sludges and soils studied were determined by the standard methods. The pH was measured potentiometrically in 1 M KCl after 24 h in the liquid/soil ratio of 10; the cation exchange capacity was determined in the 0.1 N HCl extract. The available potassium, phosphorus, and magnesium were determined according to the method described by Egner et al. (1960). The total nitrogen (N_t_) was determined by the Kjeldahl’s method (van Reeuwijk 1995) without the application of Dewarda’s alloy (Cu–Al–Zn alloy reducer of nitrites and nitrates). Total organic carbon was determined by TOC-VCSH (Shimadzu) with Solid Sample Module SSM-5000.

### Data analysis

Toxicity data were expressed as the percentage of toxic effect (PE) compared to the control. Effective concentration values (EC10 and EC50) were calculated using analysis of regression. The series of seven dilutions of the crude extract (leachates) of the sewage sludges, soil, and sewage sludges-amended soils were prepared in deionized water. The differences between each treatment and the control as well as between treatments were evaluated using a one-way analysis of variance followed by Dunnett’s post hoc test.

## Results

### Ecotoxicological characteristic of sewage sludges

Table 2 presents the values of EC10 and EC50 for both sewage sludges under study, calculated on the basis of the results obtained. Due to excessive toxicity of the sewage sludge leachates, for certain tests, it was not possible to calculate the value of EC10. In supporting information, also the effect of the sewage sludges on bacterial growth in the MARA test is presented (Fig. S1). The sludges displayed varied toxicity in relation to the test applied. Sewage sludge SL2 was characterized by greater toxicity towards *V. fischeri* than sludge SL1. A reverse tendency was noted in the test with *D. magna* and *B. calyciflorus*. The sewage sludge samples were ranked on the basis of Persoone’s et al. (2003) classification. The scoring system ranks the waters, wastewaters, or leachates in five classes of increasing hazard/toxicity, with calculation of a weight factor for the concerned hazard/toxicity class. The results obtained are presented in Table 2. Most of the tests indicated acute toxicity (class III) of investigated sewage sludges. The exception was sludge SL1 which displayed high acute toxicity (class IV) in two tests (with *D. magna* and *B. calyciflorus*). Therefore, on the basis of the above-mentioned classification (Persoone et al. 2003), it can be assumed that sludge SL1 displayed high acute toxicity and was more toxic than sludge SL2 which displayed acute toxicity.

**Table 2:** EC10 and EC50 (in percent, volume/volume) values calculated for sewage sludges used in the experiment

Organism		EC50		TU		Classification	
		SL1	SL2	SL1	SL2	SL1	SL2
*V. fischeri*	EC50	44.3 a	13.6 b	2.26 a	7.36 b	III	III
	EC10	–	–				
*T. thermophila*	EC50	46.4 a	47.9 a	2.15 a	2.09 a	III	III
	EC10	3.6 a	6.3 b				
*B. calyciflorus*	EC50	1.4 a	27.4 b	72.31 a	3.65 b	IV	III
	EC10	–	–				
*D. magna*	EC50	4.6 a	41.4 b	21.74 a	2.42 b	IV	III
	EC10	1.9 a	14.8 b				

TU = (1/EC50) × 100. Different letters between sewage sludges means that the values are significantly different

The effect of the leachates on the growth of bacteria in the MARA test varied with relation to the kind of sewage sludge. In all the cases, leachates obtained from sludge SL2 had a stimulating effect on the growth of the test organisms relative to the control. In the test with leachates obtained from sludge SL1, also their positive effect on the test organisms was observed in most cases. The exceptions were *Microbacterium* sp. (47 %), *S. warnerii* (88 %), and *P. anomala* (42 %), in the case of which a distinct toxic effect on the test organisms was noted (Fig. S1, electronic supplemental material).

### Microtox® (*V. fischeri*)

**Fig. 1:**
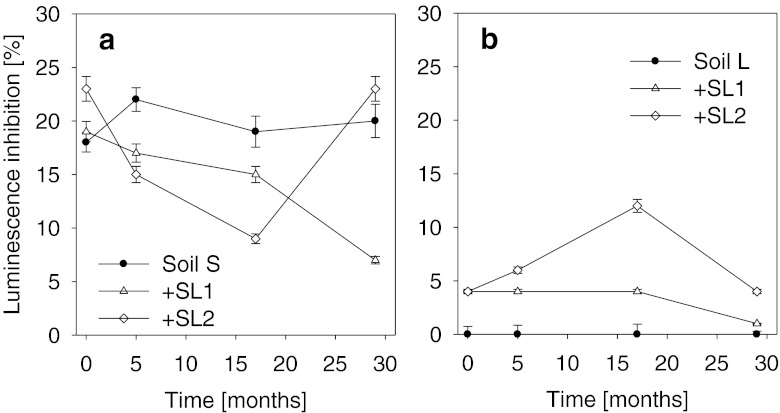
The luminescence inhibition (in percent) of leachates obtained from sewage sludge-amended soils in Microtox® bioassay. **a** Sandy soil, **b** loamy soil; *SL1 and SL2* sewage sludges. *Error bars* represent standard deviation

The addition of sewage sludges to the soils had a varied effect on their toxicity towards *V. fischeri* (Fig. 1). In soil S, only at the beginning of the experiment that a higher inhibition of luminescence was observed in the soil amended with the sludges compared to the control soil. The assayed values were statistically higher by 8.3 % (SL1) and 27.7 % (SL2) with relation to the soil without amendment with sewage sludge. On later dates of assays (after 6 and 17 months), irrespective of the sewage sludge applied, higher values of inhibition of luminescence were noted only in the control soil, which was related with significant reduction of toxicity of soils amended with the sewage sludges. During the period from the beginning of the experiment till the 17th month, in the case of both sludges, a gradual decrease of luminescence inhibition was observed. That trend persisted until the end of the experiment only in the soil amended with sludge SL1. In the case of sludge SL2, on the final date of assays, a significant increase of luminescence inhibition was noted, to the level observed in the control soil, whereas the soil amended with sludge SL1 was characterized by 65 % lower inhibition of luminescence compared to the control soil S.

Both the level of luminescence inhibition and the directions of the particular changes observed in soil L were totally different to those in soil S (Fig. 1b). Soil L had no negative effect on *V. fischeri*. The application of the sewage sludges to that soil caused a significant decrease of that parameter, indicating their increased toxicity as compared to the control soil. However, that effect was not as significant as the one observed in soil S (Fig. 1a). The values obtained on the particular dates of experiment did not exceed the level of 12 %. Immediately after the introduction in the soil, both sludges—SL1 and SL2—caused luminescence inhibition at a similar level of about 4 %. However, like in the case of soil S, also in soil L amended with the sewage sludges that further changes in the toxicity depended on the kind of sewage sludge applied. In the case of sludge SL1, inhibition of luminescence remained at a constant level until the 17th month of the study. Whereas in the case of sludge SL2, a gradual increase of toxicity of the matrix under study was observed over the same period. In both cases (sludges SL1 and SL2), the final period of the experiment was characterized by a significant decrease of the inhibition of luminescence which, in the case of sludge SL1, attained the level observed for the control soil. The soil amended with sludge SL2 continued to display a higher toxicity than that of the control soil (Fig. 1b).

### Microbial assay for toxic risk assessment

On the first date of experiment in soil S, in the case of both sewage sludges, a distinct positive effect on the test organisms was observed comparing to non-amended soil S (Fig. S2A). The range of the effect clearly depended on the type of organisms and was higher than in soil S in the range from 12.2 to 319 %. The highest values for sludge SL1 were observed for *Microbacterium* sp. (increase of 171 % comparing to the control), *S. warnerii* (319 %), and *P. anomala* (143 %), while for sludge SL2, values above 100 % were observed for *C. testosteroni* (124.2 %) and *S. warnerii* (227.2 %). The lowest effect of the addition of the sewage sludges was noted in the case of *P. aurantiaca* (13.5 %) and *S. rubidaea* (12.2 %) in sludge SL1, and *Microbacterium* sp. (20.4 %) and *S. rubidaea* (0 %, no change) in sludge SL2. With the passage of time, all the test organisms displayed the same tendency in soil S amended with sludge SL1. During the period from the beginning of the experiment till the 17th month of the study, a gradual decrease was observed in the positive effect of the soil amended with sludge SL1. In the case of one third of the test organisms, i.e., *C. freundii*, *K. gibsonii*, *P. aurantiaca*, and *S. rubidaea*, the values assayed on the third date of analyses were lower than those observed on the corresponding dates in soil S, by 46.6, 32.8, 25.0, and 36.5 %, respectively. On the final date of assays, another increase of the positive effect of the sludges on all test organisms was noted. That increase attained values similar to or higher than the level observed directly after the addition of the sewage sludges. The effect of sludge SL2 on the toxicity of soil S over time had a completely different way. In the case of *Microbacterium* sp., *S. warnerii*, *S. rubidaea*, and *P. anomala*, throughout the period of the experiment, a gradual increase of positive effect of SL2 on the amended soil was observed. Whereas in the case of four bacteria strains (*B. diminuta*, *C. freundii*, *D. acidovorans*, and *P. aurantiaca*), no significant changes were noted between the particular dates of assays. For all of the test organisms, the values determined on the final date of analyses were higher, by from 23 to 52 %, than the values observed in the control soil at the same time.

The addition of sludges SL1 and SL2 to soil L had a more diversified character than it was observed in the case of soil S. The direction of the effect of the sludges was also less positive in character than it was noted in soil S. The application of both sludges to soil L caused a reduction of growth in the case of nearly one half of the organisms tested (*Microbacterium* sp., *D. acidovorans*, *S. warnerii*, *S. rubidaea*, and *P. anomala*). The greatest rates of growth reduction, amounting to 51 and 57 %, were noted in the case of organisms *S. warnerii* and *P. anomala*, respectively. A positive effect of soil amendment with the sludges was found only for *C. freundii* (SL1 and SL2) and *C. testosteroni* (SL1). In the case of the other test organisms, no significant effect of the sludges on their growth was noted relative to the control soil L. Values determined at the beginning of the experiment in soil L fertilized with sludge SL1 remained at a constant level for all the test organisms throughout the period of the experiment. In no case, statistically significant differences were noted between the dates of assays, whereas a greater variation between the dates of assays was observed in soil L with an addition of sludge SL2 (Fig. S2B). In the case of most of the test organisms (*B. diminuta*, *C. freundii*, *D. acidovorans*, *C. testosteroni*, *E. casseliflavus*, and *K. gibsonii*), the same tendency was observed as in the control soil. It consisted in a gradual increase of toxicity up till the third date of assays, and on the final date of analyses, another increase was noted (more significant), usually up to the level observed at the beginning of the experiment. From the beginning to the end of the study, a gradual increase of positive effect of the sludges on organisms, *Microbacterium* sp., *S. rubidaea*, and *P. anomala*, was observed.

### Prototoxkit F™ (*T. thermophila*)

**Fig. 2:**
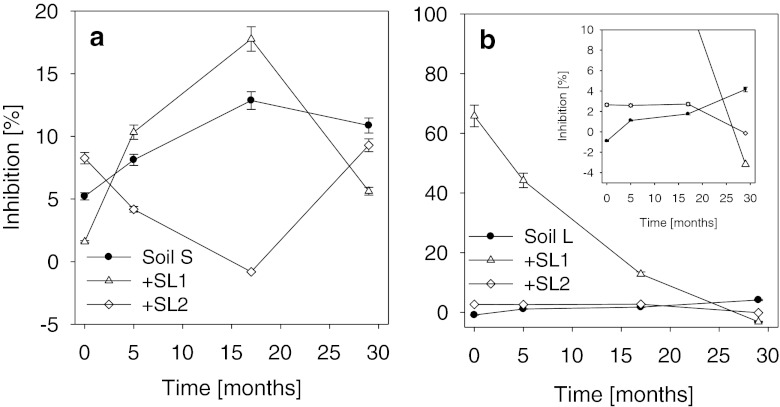
Inhibition of *T. thermophila* (Prototoxkit F™) by leachates obtained from sewage sludge-amended soils. **a** Sandy soil, **b** loamy soil; *SL1 and SL2* sewage sludges. *Error bars* represent standard deviation

As in the case of *V. fischeri*, the effect of the sewage sludges on the toxicity of soils towards *T. thermophila* depended to a significant degree on the type of soil and on the kind of sewage sludge (Fig. 2). The addition of the two kinds of sewage sludge to soil S had a completely different effect on the toxicity of the amended soil towards *T. thermophila*. Subsequent changes in the toxicity were also related to the kind of sewage sludge. At the beginning of the experiment, the addition of sludge SL1 caused a significant decrease in the toxicity of soil S as compared to the soil with no sludge amendment (by 69.5 %), whereas sludge SL2 caused a distinct negative effect at the level of 8.3 % (higher by 58.5 % in relation to soil S without amendment with the sludge). For soil amended with sludge SL1, a gradual increase of its toxicity was observed until the third date of experiment. That toxicity, both on the second and third dates of experiment, exceeded the level of toxicity observed for soil S with no amendment with the sludge. During the final 12 months of the experiment (from the 17th to the 29th month), a rapid decrease of toxicity was noted, to a level below the value recorded for the control soil (Fig. 2a). In the case of sludge SL2, as mentioned above, a reverse tendency was observed. In spite of the initial negative effect observed after the amendment of soil S with sludge SL2, subsequently, a gradual decrease of toxicity was noted, and already on the second date of assays, its level was lower than that in the control soil by 48.5 %. That decreasing trend continued until the third date of analyses, after which a violent increase of toxicity was observed. Ultimately, for both kinds of sewage sludge, on the final date of assays, the level of toxicity of soils amended with SL1 and SL2 was below the level observed for the control soil (Fig. 2a).

The addition of sludge SL2 to soil L had only a slight (but statistically significant, *P ≤* 0.05) increasing effect on its toxicity towards *T. thermophila* (Fig. 2b). Inhibition of the test organisms greater than that in the control soil continued until the 17th month of the experiment, following which a significant decrease of the toxicity was observed, to below the level observed in the control soil L (Fig. 2b), whereas a significant effect was observed in the case of sludge SL1 which, already from the first date of experiment, caused significant inhibition of *T. thermophila* at the level of 64 %. With the passage of time, however, a gradual decrease of the toxicity was observed. On the final date of analyses, as in the case of sludge SL2, the value of inhibition of *T. thermophila* was lower than that observed for the control soil L.

### Rotoxkit F™ (*B. calyciflorus*)

**Fig. 3:**
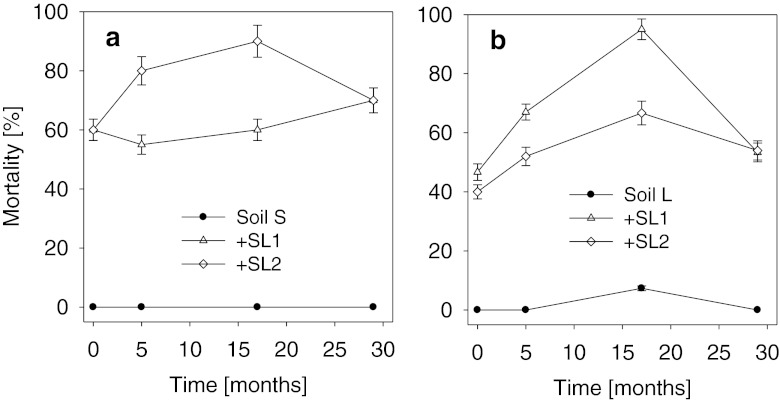
Mortality of *B. calyciflorus* (Rotoxkit F™) by leachates obtained from sewage sludge-amended soils. **a** Sandy soil, **b** loamy soil; *SL1 and SL2* sewage sludges. *Error bars* represent standard deviation

The addition of the sewage sludges to soils S as well as to soil L caused significant mortality of *B. calyciflorus* (Fig. 3). At the beginning of the study, that effect was more pronounced in soil S than in soil L. It was also observed that the toxicity of the particular sludges with relation to the test organisms differed in relation to the type of soil. In soil S, greater toxicity (except for assay dates I and IV) was a characteristic of sludge SL2, while in soil L, almost throughout the period of the study (except for the final date of study), sludge SL1 displayed higher toxicity than sludge SL2. Like in the case of organisms tested earlier, the change of toxicity clearly depended on the soil type, though to a lesser degree than on the sewage sludge. In soil S for sewage sludge SL2 and in soil L in the case of sludges SL1 and SL2, an increase of toxicity was noted from the beginning of the study. That tendency persisted until the 17th month of the experiment, following which a significant decrease of toxicity was noted in the case of both sludges, to a level similar to that from the beginning of the experiment (Fig. 3). Only in soil S amended with sludge SL1 that a slight decrease of toxicity was observed on the second date of experiment, followed by a gradual increase which continued until the end of the experiment (Fig. 3).

### Daphtoxkit F™ (*D. magna*)

**Fig. 4:**
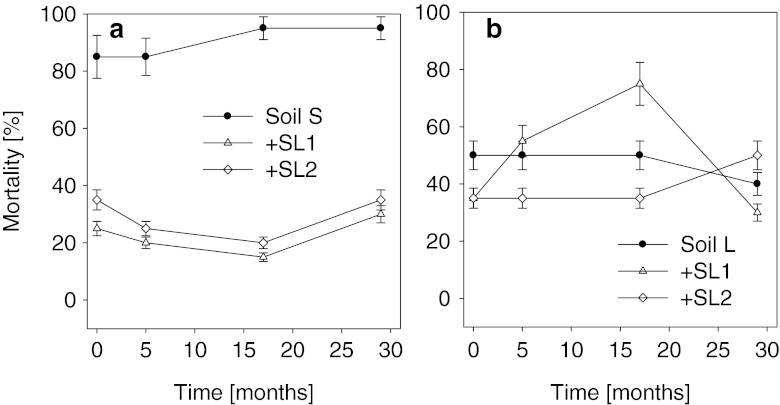
Mortality of *D. magna* (Daphtoxkit F™) by leachates obtained from sewage sludge-amended soils. **a** Sandy soil, **b** loamy soil; *SL1 and SL2* sewage sludges. *Error bars* represent standard deviation

Figure 4 presents the effect of the sewage sludges on the toxicity of the soils towards *D. magna*. Leachates obtained from the control soil S were characterized by high toxicity with relation to *D. magna*. Depending on the date of analyses, the rate of mortality of *D. magna* in that soil varied from 85 to 95 %. The addition of sludges SL1 and SL2 to soil S had a positive effect on *D. magna*, causing a decrease of its toxicity by more than a half. A slightly better effect was noted for sewage sludge SL1 than SL2 (Fig. 4a). Totally different results were obtained in the case of soil L amended with the sewage sludges. Although on the first date of assays the addition of the sludges to soil L had a positive effect on *D. magna* (Fig. 4b), in the case of sludge SL2, that trend continued only until the 17th month of the experiment. After that time, a significant increase of toxicity was noted, to above the level observed for the control soil L. The toxicity in soil L fertilized with sludge SL1 changed in a different manner, with a gradual increase over the initial 17 months. It was only in the period from the 17th to the 29th month of the experiment that a significant decrease of toxicity was observed in soil L amended with sludge SL1.

### EC10 values at the beginning and the end of the experiment

To permit comparison of the sensitivity of the particular test organisms with relation to the materials under study, the values of EC10 were determined on the basis of a series of dilutions of the raw leachates. Due to the low toxicity of most of the leachates, the calculation of the values of EC50 was not possible. The values of EC10 were determined for samples taken at the beginning of the experiment and on the final stage of study. The results obtained are presented in Table 3. *T. thermophila* proved to be the least sensitive organism, both at the beginning and at the end of the experiment. Due to the fact that in certain cases PE < 10 %, it was impossible to calculate even the value of EC10 for some of the variants of the experiment. The most sensitive organism to the presence of the sewage sludges proved to be *B. calyciflorus*. For that organism, the values of EC10, depending on the date of assays, soil type, and kind of sewage sludge, varied from 4.4 to 23.8 %, displaying greater toxicity in soil S than in soil L (Table 3). In the case of *D. magna*, EC10 also assumed relatively low values, but that resulted primarily from the fact of high toxicity towards those organisms in non-amended soils S and L and, probably, was not a result of the presence of the sewage sludges. It is noteworthy that the addition of the sludges caused a considerable decrease of the negative effect of the soils under study on *D. magna* (Table 3). In the case of *V. fischeri*, the values of EC10 were determined only in soil S and in soil S amended with the sewage sludges. This was due to either zero effect or PE < 10 % in soil L and in soil L amended with the sewage sludges. The hierarchy of the levels of sensitivity of the particular organisms after the addition of sludges SL1 and SL2 to soil S was identical: *T. thermophila* < *V. fischeri* < *D. magna* < *B. calyciflorus*. Whereas in soil L, differences observed between the sludges were as follows: SL1, *V. fischeri* < *D. magna* < *B. calyciflorus* < *T. thermophila*, and SL2, *T. thermophila* = *V. fischeri* < *B. calyciflorus* < *D. magna*.

**Table 3:** EC10 (in percent, volume/volume) values determined for leachates received at the beginning (I) and the end (IV) of experiment from soils (S and L) and sewage sludge-amended soils (+SL1 and +SL2)

Organisms	Soil S			+SL1			+SL2			Soil L			+SL1			+SL2		
	I	IV	%	I	IV	%	I	IV	%	I	IV	%	I	IV	%	I	IV	%
*V. fischeri*	45.5	34.3	24.6	42.6	>100	−	29.9	33.5	−11.8	NTE	NTE	NC	>100	>100	NC	>100	>100	NC
*T. thermophila*	>100^a^	85.2	*+*	>100	>100	NC	96.8	82.5	14.8	NTE	>100	*+*	11.4	>100	*−*	>100	NTE	*−*
*B. calyciflorus*	NTE	NTE	NC	12.3	6.3	48.7	12.3	4.4	64.3	NTE	NTE	NC	17.0	10.0	41.2	23.8	17.4	27.0
*D. magna*	8.2	8.3	−1.6	30.3	22.4	26.1	21.0	19.1	9.2	13.1	17.1	−42.3	29.4	33.3	−13.3	22.1	14.9	32.5

I and IV are samples from beginning and the end of the experiment; percent change (positive values (+) means increasing of the toxicity, and negative values (−) means decreasing of the toxicity)

*NTE* no toxic effect, *NC* no change

^a^Values higher than 100 % because of PE < 10 % for raw leachate

Analyzing the effect of time on the values of EC10, a notable increase of toxicity on the final date of study relative to the beginning of the experiment was observed in the case of *B. calyciflorus*. The range of a decrease of EC10 (increase of toxicity) in relation to the beginning of the experiment varied from 48.7 to 64.3 % in soil S and from 27.0 to 41.2 % in soil L, depending on the kind of sewage sludge. In three out of four cases, an increase of toxicity after 29 months of the experiment was observed on soil amended with the sludge also for *D. magna*. In the case of the remaining two tests, more often, a lack of change or a decrease of toxicity was observed than its increase.

## Discussion

In spite of the lack of legislative regulations concerning the possibility of applying biological tests for the estimation of toxicity of sewage sludges, that method is more and more frequently applied. This results primarily from the fact that biological tests permit more precise determination of toxicity than chemical analyses, indicating directly the potential threats. Studies conducted so far have been mainly focused on the estimation of toxicity of sewage sludges (Fuentes et al. 2006; Hu and Yuan 2012; Oleszczuk 2008, 2010; Ramirez et al. 2008b; Roig et al. 2012), without taking into account such important parameters as the type of soil in which a sewage sludge is applied, or the varied effect of sewage sludges on various organisms. In the case of the latter, it is especially important to apply a battery of biotests that will permit more comprehensive determination of potential threats related with sewage sludge with respect to various organisms. Studies of this type, sparse so far, are focused mainly on effluents from sewage treatment plants (Latif and Licek 2004; Manusadzianas et al. 2003) or landfill leachates (Clement et al. 1996; Matejczyk et al. 2011). Leachates obtained from the sewage sludges used in this study displayed varied levels of toxicity towards the test organisms (Table 2). The addition of the sludges to the soil significantly reduced their toxicity, which was related with the “dilution” of the sludges with the soils studied. Although the sludges differed from each other in the level of toxicity, no identical trends were found after the introduction of the sludges to the soils. For example, sludge SL2 which displayed higher toxicity than sludge SL1 in the Microtox test (Table 2) had a lesser negative effect on *V. fischeri* after the introduction in the soil (Table 3) than sludge SL1. A similar tendency was observed with relation to the other test organisms. That phenomenon is hard to explain. Most probably, it is related to the multidirectional effect of various factors (mainly the properties of the soils and their components) which, after the application of the sludges, may have various increasing or decreasing effects on toxicity. Undoubtedly, more detailed elucidation of the problem is the key element in further studies on the toxicity of soils amended with sewage sludge. Only in the case of *B. calyciflorus* that the difference in toxicity between sludges (greater toxicity of sludge SL1 than SL2) was confirmed also after the introduction of the sludges in both of the soils, which indicates that the species may be a potential indicator of toxicity of sewage sludges. However, it should be emphasized that the capacity of *B. calyciflorus* as an indicator has been tested here only for the two soils. It might behave differently in a different type of soil.

The study demonstrated clearly that the effect of sewage sludges on the toxicity of soils may vary in relation to the biological test applied. This is understandable taking into account that various organisms can be characterized by various levels of sensitivity to particular pollutants (Isidori et al. 2006). In no case, any significant relationships were noted among the results obtained for the particular tests, which indicates diversified sensitivity of the test organisms. This confirms the species-specific as well as chemical-specific nature of toxicity, reflected by the different impacts on different test species (Clement et al. 1996; Latif and Licek 2004). It is difficult to relate the results obtained to the results of research by other authors, due to the lack of publications pertaining to this subject matter. Earlier studies by Matejczyk et al. (2011) showed that such a relationship can occur. However, it usually concerns a single group of organisms, e.g., crustaceans, and is rarely observed in relation to various groups of organisms. This supports the necessity of research on sewage sludges in the aspect of their effect on various organisms to achieve a full characterization of threats.

Studies conducted so far on the toxicity of soils amended with sewage sludges have been concerned mainly with earthworms (Cesar et al. 2012; Natal-da-Luz et al. 2009a), collembola (Domene et al. 2007; Natal-da-luz et al. 2009b), or plants (Oleszczuk et al. 2012). Those studies were mostly focused on the estimation of the solid fraction of sewage sludge. Studies on leachates, as mentioned before, are focused on other environmental matrices. However, leachates can be useful for evaluating toxicity of sewage sludge-amended soils on aquatic organisms in hypothetic situations where soil leaching and erosion processes could mobilize contaminants to surrounding fluvial systems (Baun et al. 2002; Cesar et al. 2012). In the study presented here, the highest sensitivity to the presence of sewage sludge in soil S was a characteristic of *B. calyciflorus*. That species is relatively rarely used in ecotoxicological studies (Isidori et al. 2003; Matejczyk et al. 2011). Rotifers, however, are an important group of organisms which plays an important role in the functioning of aquatic ecosystems (Ejsmont-Karabin 2012). As demonstrated by results obtained so far (Isidori et al. 2003), rotifers are characterized by greater sensitivity than *D. magna*, which found support also in the study presented here, whereas Matejczyk et al. (2011) observed a reverse tendency, indicating greater sensitivity of *D. magna* than *B. calyciflorus* to landfill leachates. In this study, a similar relation was observed only in soil L amended with sludge SL2. This indicates clearly that the effect of sewage sludges on toxicity may vary radically not only in relation to the kind of sludge (and probably the contaminants contained in it) but also to the type of soil. Relatively, the least sensitive organism in this study proved to be *T. thermophila* (with the exception of soil L with SL1), which was also supported in other studies (Latif and Licek 2004), whereas rather surprising is the low sensitivity of the bacteria *V. fischeri*. *V. fischeri* inhibition (Microtox® assay) is widely accepted as a good indicator of the environmental impact of certain wastes and leachates produced by human activity (Roig et al. 2012). In this study, the sensitivity of that test, compared to others, placed it in the group of the less sensitive ones, which again confirms the necessity of expanding the scope of research onto other groups of organisms.

As follows from the results obtained, the type of soil in which sewage sludge is introduced is an especially important parameter. The results are in agreement with other tests conducted with natural soils, suggesting that soil properties played a vital role in the toxicity (Cesar et al. 2012; Domene et al. 2010; Oleszczuk et al. 2012). In the study presented here, the sensitivity of the particular test organisms was clearly dependent on the soil type. It can be assumed that it was the factor that played the most important role in the variation of toxicity. Also, the changes of toxicity on the particular dates of assays were determined by that parameter. More frequently, the sewage sludges were characterized by greater toxicity when introduced into soil S than into soil L. This is understandable, taking into account the physicochemical properties of the soils studied and their particle size distribution. Soil S was characterized by relatively weak fertilizing properties (Table 1); moreover, the sorption complexes (CEC) and content of clay which could be responsible for bioavailability and mobility of organic and inorganic pollutants (Heemsbergen et al. 2009) were also unfavorable in soil S. Clay minerals in the soil L may have stimulated the reduction of metal contents in the soil solution comparing to soil S, due to its high potential of cationic adsorption (Cesar et al. 2012). Cesar et al. (2012) found lower levels of toxicity associated with the presence of expensive clay minerals, thus suggesting that those minerals are able to decrease not only the mobility of contaminants in the environment but also the toxicity to earthworms. It should also be emphasized that soil S was characterized by low pH (Table 1), which could cause, after the introduction of the sludges, mobilization of heavy metals and their increased transfer to the leachates. It is well-known that pH can influence the oxidation and mobility of metals in the soil, affecting their bioavailability and toxicity (Alkorta et al. 2006; Cesar et al. 2012).

In the estimation of the toxicity of sewage sludges, it is extremely important to determine their toxicity not only immediately after their introduction into soil but also at later times (Natal-da-luz et al. 2009b; Oleszczuk et al. 2012). As demonstrated by the results of this study, the potential toxicity can undergo significant changes. This problem, however, is so far the least elucidated in the literature. The few studies conducted so far (Natal-da-luz et al. 2009b), in which sewage sludges together with soil were incubated for periods of 0, 4, and 12 weeks, showed that the toxicity of sludges can be subject to variation. The range of the variation depended on the kind of sludge and on the parameter tested (avoidance or reproduction of *Folsomia candida*). Lowering of the toxicity of soils amended with sewage sludges is most probably the result of one of the following factors: (1) biodegradation, (2) sequestration or formation of bound residue contaminants, (3) leaching of contaminants or their uptake by plants, and (4) change of the physicochemical conditions of soils, leading to a decrease or an increase in the intensity of the above-mentioned processes. An example in this case can be the mineralization of organic matter. That process, on the one hand, leads to increased stability of organic matter and to enhancement of its sorptive capacity to organic contaminants (reduction of toxicity as a result of limitation of the mobility of contaminants) (Oleszczuk 2009). On the other hand, however, mineralization of organic matter (26–42 % of the organic matter introduced together with the sewage sludge underwent mineralization very quickly) (Rowell et al. 2001) from sewage sludge may result in the liberation of contaminants that were hard available before, intensifying their negative effect on the test organisms. The phenomenon of increase of toxicity, especially in the case of *B. calyciflorus* but also of other test organisms (Table 3), was observed on the final date of assays relative to the beginning of the experiment. It is also important to emphasize that the positive effect of the sewage sludges on the test organisms observed initially frequently changed to a negative effect on the later dates of analyses. This was particularly observable in the MARA test, where the leachates from the sewage sludges displayed a positive effect on the growth of the test organisms, which appeared also after the addition of the sludges to the soil. On the one hand, that could have been related with the mobilization of contaminants, as a result of the processes mentioned earlier, but on the other hand, the mineralization of organic matter, constituting a source of nutrients for microorganisms, as well as the leaching of nutrients or reduction of their availability from soil amended with the sludges compared to the beginning of the experiment, notably inhibited the growth of those organisms in later periods, which resulted in a reduction of their activity. No studies making use of the MARA test for the estimation of toxicity of soils fertilized with sewage sludges have been presented so far. As can be seen from the study presented here, the effect of sewage sludges is varied, but as in the case of the preceding tests, it is determined by the soil type to a significant degree.

## Conclusion

To our knowledge, the research results presented here are the first to be obtained in a study in which a battery of biotests has been applied for the estimation of the toxicity of soils amended with sewage sludges in a long-term approach. The results show that the addition of sewage sludges to the soils has a significant effect on their toxicity which, depending on the test organism and soil type, may assume various values. The differences between the soils and among the test organisms can vary within very broad ranges. However, the results show clearly that *B. calyciflorus* was the organism that was characterized by the highest sensitivity to the presence of the sewage sludges. What is interesting with the passage of time is the toxicity of the soils amended with the sewage sludges increased both in the case of *B. calyciflorus* and of some of the variants with other test species. This indicates the need of estimations of sewage sludges, not only taking into account the soil type, as suggested before, but also of applying the long-term approach to the problem. The most popular in ecotoxicological estimations test with the use of the bacteria *V. fischeri* proved to be one of the least sensitive. A low level of sensitivity with relation to bacteria has also been found in the case of the MARA test which, in a majority of cases, indicated a positive effect of the sewage sludges. A low level of sensitivity to the presence of sewage sludges was also displayed by *T. thermophila*. Bacteria and protozoans play a very important role in the self-purification of waters. Contaminants affecting those organisms may have an indirect effect on the transformation of pollutants in waters and soils. Therefore, the demonstration of a slight effect of sewage sludges on those groups of organisms constitutes a positive aspect in the context of utilization of sewage sludges. However, it should be emphasized that without a doubt, as indicated by our study, the estimation of the toxicity of sewage sludges should be based on a comprehensive approach to the problem, taking into account not only various groups of test organisms but also an estimation of the effect of the type of soil into which the sludge is introduced and of the time elapsed since the application of sewage sludge.

## Electronic supplementary material

Below is the link to the electronic supplementary material.

ESM 1 (DOC 1745 kb)
