# Supplementary material for: Application of a battery of biotests for the determination of leachate toxicity to bacteria and invertebrates from sewage sludge-amended soil
Source: Environ Sci Pollut Res Int. 2012 Nov 7;20(5):3435–46. doi: 10.1007/s11356-012-1268-3 (PMC3633785; doi:10.1007/s11356-012-1268-3)
Supplement: Supplementary file 1 — (DOC 1745 kb) [file 11356_2012_1268_MOESM1_ESM.doc]

Supporting information:

**APPLICATION OF BATTERY OF BIOTESTS FOR DETERMINATION OF SEWAGE SLUDGE-AMENDED SOIL LEACHATES TOXICITY TO BACTERIA AND INVERTEBRATES**

Anna Malara, Patryk Oleszczuk^*^

*Maria Curie-Skłodowska University, Faculty of Chemistry, 3 Maria Curie-Skłodowska Square, 20-031 Lublin, Poland*

*Corresponding author: Patryk Oleszczuk (*patryk.oleszczuk@up.lublin.pl*)

Journal: *Environmental Science and Pollution Research*

Number of pages: 3

Number of figures: 3 (Fig. 1 and Fig. 2A and 2B)

Figure S1. Effect of sewage sludge leachates on microorganisms growth in MARA test. 1 – *Microbacterium* sp., 2 – *Brevundimonas diminuta*, 3 – *Citrobacter freundii*, 4 – *Comamonas testosteroni*, 5 – *Enterococcus casseliflavus*, 6 – *Delftia acidovorans*, 7 – *Kurthia gibsonii*, 8 – *Staphylococcus warnerii*, 9 – *Pseudomonas aurantiaca*, 10 – *Serratia rubidaea*, 11 – *Pichia anomalya.* Error bars represents standard deviation.

**Figure S2A.** Effect of sewage sludges-amended soil S leachates on bacteria and yeast determined by MARA method. SL1 and SL2 – sewage sludges. Error bars represents standard deviation. 1 – *Microbacterium* sp., 2 – *Brevundimonas diminuta*, 3 – *Citrobacter freundii*, 4 – *Comamonas testosteroni*, 5 – *Enterococcus casseliflavus*, 6 – *Delftia acidovorans*, 7 – *Kurthia gibsonii*, 8 – *Staphylococcus warnerii*, 9 – *Pseudomonas aurantiaca*, 10 – *Serratia rubidaea*, 11 – *Pichia anomalya.* Error bars represents standard deviation.

**Figure S2B.** Effect of sewage sludges-amended soil L leachates on bacteria and yeast determined by MARA method. SL1 and SL2 – sewage sludges. 1 – *Microbacterium* sp., 2 – *Brevundimonas diminuta*, 3 – *Citrobacter freundii*, 4 – *Comamonas testosteroni*, 5 – *Enterococcus casseliflavus*, 6 – *Delftia acidovorans*, 7 – *Kurthia gibsonii*, 8 – *Staphylococcus warnerii*, 9 – *Pseudomonas aurantiaca*, 10 – *Serratia rubidaea*, 11 – *Pichia anomalya.* Error bars represents standard deviation.
